# Changes in practitioners’ attitudes, perceived training needs and self-efficacy over the implementation process of an evidence-based parenting program

**DOI:** 10.1186/s12913-020-05939-3

**Published:** 2020-11-27

**Authors:** Marie-Kim Côté, Marie-Hélène Gagné

**Affiliations:** Université Laval, 2325 rue de l’Université, Québec, QC G1V 0A6 Canada

**Keywords:** Implementation science, Practitioners, Attitudes, Self-efficacy, Organizational capacity, Evidence-based program, Triple P – positive parenting program

## Abstract

**Background:**

Evidence-based family support programs such as the Triple P – Positive Parenting Program have the potential to enhance the well-being of children and families. However, they cannot achieve their expected outcomes if insufficient attention is paid to the implementation process. It has been demonstrated that practitioners’ attitudes towards evidence-based programs (EBPs), perceived training needs and self-efficacy for working with parents influence implementation outcomes (e.g., program acceptability, adoption, adherence and sustainability). At the same time, the experience of being involved in the implementation process of an EBP could enhance practitioners’ perceptions of the initiative. This study aimed to assess changes in practitioner’s attitudes, perceived training needs and self-efficacy over a two-year EBP implementation process, in interaction with their appraisal of their organization’s capacity to implement the EPB.

**Methods:**

In the province of Quebec, Canada, Triple P was implemented and evaluated in two communities. Ninety-nine practitioners from various organizations completed questionnaires shortly before their training in Triple P and two years later.

**Results:**

Findings show that practitioners who displayed more initial skepticism regarding their organization’s capacity to implement the program reported greater improvements in attitudes over time, while practitioners who showed more optimism at baseline reported a greater decrease in their perceived training needs. Practitioners’ self-efficacy increased moderately regardless of perceived organizational capacity.

**Conclusions:**

These results are encouraging given that more positive perceptions of EBPs could foster the systematic use of these programs in communities, for the potential benefit of a greater number of families.

**Supplementary Information:**

The online version contains supplementary material available at 10.1186/s12913-020-05939-3.

## Background

It is increasingly recognized that choosing empirically supported interventions is not enough to improve the well-being of children and families. The way evidence-based programs (EBPs) are implemented within existing delivery systems also matters. Implementation is considered the “missing link” between research and practice [1]. According to Durlak and Dupre [2], the mean effect size of a program’s outcomes can be up to twelve times higher when ideal implementation conditions are met. Optimal program outcomes are contingent on the achievement of implementation outcomes such as program adoption (intention to try), adherence (program delivered as intended) and sustainability (sustained use) [3].

Practitioners’ attitudes towards EBPs, perceived training needs and self-efficacy for working with parents, in particular, have been shown to predict these positive implementation outcomes [4, 5]. At the same time, the experience of being involved in the implementation of an EPB could change practitioners’ perceptions regarding the relevance of EBPs in their practice, potentially reducing their resistance to these programs, sometimes considered a major barrier to the systematic adoption of EBPs in communities [6, 7]. This study thus aims to document changes over time in the attitudes, perceived training needs and self-efficacy of practitioners involved in the implementation of an evidence-based parenting program, namely the Triple P – Positive Parenting Program.

Attitudes, both affective and cognitive in nature, play an important role in orienting people’s decisions and behaviors [8, 9] and are essential components of many motivational theories [10]. Drawing on these theoretical perspectives, many authors have investigated practitioners’ attitudes as predictors of implementation outcomes [11, 12]. Studies have shown that favorable attitudes towards EBPs are related to program adherence [4], commitment to and satisfaction with EBP training, and subsequent use of EBPs [13]. Various factors also appear to influence the valence and intensity of practitioners’ attitudes towards EBPs, such as their prior knowledge of these programs [14], level of education and amount of previous experience as clinicians [15].

Practitioners’ perceived training needs related to their intervention abilities are considered a dimension of motivational readiness for change, according to Simpson’s [16] conceptual framework for transferring research into practice. When practitioners perceive that they could benefit from further training to enhance their work with clients, they may be more inclined to bring about changes in their practice. One way of achieving this could be to engage practitioners in the implementation process of an EBP. Higher motivational readiness, measured, for example, among practitioners in the field of treatment addiction, through a combination of perceived pressure to change and perceived training needs, has been linked to increased adherence to the core components of a cognitive-behavioral EBP [17].

According to Bandura [18], self-efficacy refers to a person’s confidence in his/her capability to perform a specific task and is thought to have a greater influence on actual behavior and performance than the person’s objective ability to do the task [19]. In the implementation field, self-efficacy refers to the degree of practitioners’ confidence in their ability to deliver the program components. When it comes to delivering evidence-based parenting programs in particular, practitioners’ level of self-efficacy is considered an important predictor of implementation outcomes such as increased program use [5, 20] and increased ability to deliver the program with both flexibility and fidelity to its core components [21]. Mazzucchelli and Ralph [22] conceptualize self-efficacy (the capacity to undertake specific therapeutic tasks) as a component of self-regulatory (the ability to manage one’s own emotions and behaviors to achieve specific goals), along with self-management (the capacity to define and monitor goals for himself and the client), personal agency (the tendency to attribue changes to clients and their own efforts instead of chance), self-sufficiency (the ability to be an independent problem solver who also seek support when needed) and problem solving (the capacity to define a problem and select strategies to overcome it). According to these authors, self-regulatory skills in practitioners are crucial, because it allows them to change their own behavior in response to cues and information about the current needs of parents to be more effective when working with them, independantly of the organizational culture or context [22].

While practitioners’ attitudes towards EBPs, perceived training needs and self-efficacy have mainly been examined as determinants of a program’s efficacy or effectiveness, few studies have investigated changes in these variables over time, particularly in the child and family services field. Regarding attitudes, Lim et al. [14] observed an increase in the appeal of EBPs as perceived by community mental health practitioners immediately following their participation in three workshops on evidence-based techniques intended to decrease internalizing and externalizing problems among youths. Another study involving five measures over a 14-month period yielded different results, with no changes in attitudes towards EBPs being observed among practitioners in the child welfare sector who received training in an evidence-based parenting program [23]. However, as pointed out by the authors of this study, the study context did not involve a “full implementation strategy.” Had such a strategy been applied, it is possible that training in this program would have had a greater impact on participants’ attitudes. The same limitation applies to a study conducted over a two-year period in which no changes were found in perceived training needs among substance abuse treatment counselors who participated in a workshop on EBPs at mid-point in the study [24]. However, being trained in an EBP appears to have a significant positive effect on practitioners’ self-efficacy for delivering the program’s components. Studies in various fields, such as the promotion of healthy habits among children [25] and parenting skills training [5, 26], have demonstrated this effect, with the interval between measures ranging from a few days to more than two years.

In summary, studies examining the evolution of practitioners’ attitudes, perceived training needs and self-efficacy have yielded mixed results. Moreover, most of these studies did not take place in a context where practitioners participated in a structured implementation process. Such a process involves multiple steps requiring sensitivity to the context and the point of view of actors in the field [27]. Among the many frameworks describing the steps or stages of implementation [28], the present study used the *Quality Implementation Framework* (QIF) as the basis for the hypotheses formulated. The QIF was developed by Meyers, Durlak, and Wandersman [29] by synthesizing 25 previous models. It conceptualizes the implementation process in terms of fourteen steps, such as conducting a fit assessment between the host setting and the chosen program, recruiting and training staff, and creating an ongoing monitoring system to provide technical assistance and supportive feedback. The last step of this process is labeled “learning from experience.” The QIF is a cyclical model based on the assumption that the experience gained through the implementation process of any EBP will lead to new learning. This learning will be useful for building organizational capacity (i.e., resources, competencies, attitudes, coordination, etc.) and can later be generalized to start a new cycle of implementation [29]. This assumption suggests that being part of a structured implementation process will have a greater impact on practitioners’ attitudes, perceived training needs, and self-efficacy than mere exposure to an EBP training program. In any case, as pointed out by Weisz et al. [30], given all the efforts that have been put into EBP implementation processes, it is now important to focus on practitioners’ responses to them.

An investigation of this nature should take into account the organizational context in which the implementation of an EBP takes place. Although implementation models typically emphasize the role of both provider- and organization-level factors [31, 32], little is known regarding the interaction between these two levels of factors in the implementation process of EBPs [33, 34].

An organization’s capacity to implement an EBP involves multiple aspects such as administrative support, funding, the clarity of the agency’s mission and goals, staff and supervisor buy-in, staff cohesion, and the quality of clinical supervision [35–37]. Practitioners’ subjective appraisal of these organizational factors has been linked to their attitudes, perceived training needs and self-efficacy. For instance, Izmirian and Nakamura [38] found that practitioners in youth mental health services were more likely to have positive attitudes towards EBPs when they reported experiencing a less stressful work environment. Nurses’ attitudes towards EBPs were also found to improve following an organizational intervention that included mentoring by a nurse researcher and the provision of funding to attend conferences promoting the use of evidence-based practices [39]. Moreover, having a supervisor that promotes teamwork and cohesion has been linked to higher levels of self-efficacy, especially among practitioners with less than two years’ experience in their field [19]. Finally, employees’ level of trust in their organization (defined as positive expectations regarding organizational support, integrity and consistency) has been found to moderate the influence of self-efficacy on job satisfaction and task performance [40].

In light of these considerations, the present study aimed to assess changes in practitioners’ attitudes towards EBPs, perceived training needs, and self-efficacy for working with parents over a two-year EBP implementation process. Based on the aforementioned studies and the assumption of the QIF that any implementation process will generate new learning [29], we expected to see an improvement in attitudes, an increase in self-efficacy, and a decrease in perceived training needs over time. It was hypothesized that the level of change in all these variables would be moderated by the level of confidence in the organization’s capacity to implement the program at baseline (i.e., a higher subjective rating of organizational capacity would lead to a greater improvement in attitudes, a greater increase in self-efficacy and a greater decrease in perceived training needs).

## Methods

### Context

This study is part of a larger study evaluating the implementation of an EBP, the Triple P – Positive Parenting Program, in two health services catchment areas in the province of Quebec, Canada. Triple P entails a five-level integrated system of universal, selective, and indicated interventions whose intensity increases along with the needs of parents of 0–12 year-old children [41]. There is significant scientific evidence supporting Triple P’s efficacy for increasing positive parenting practices and reducing emotional and behavioral problems in children [42–45]. There is also some evidence that Triple P prevents child maltreatment [46, 47]. The present study focused on Selective Triple P (Level 2 – public seminars), Primary Care Triple P (Level 3 – individual coaching), Group Triple P (Level 4 – parent training), and Pathways Triple P (Level 5 - active skills training including cognitive reattribution components), delivered by trained practitioners [41]. Service delivery was supported by a promotional campaign (Level 1) developed locally [48, 49]. In each community implementing Triple P, a team of community partners carefully planned the implementation process [50]. These partners came from different sectors of activity (child care services, schools, non-governmental and governmental organizations). Managers in the partner organizations targeted practitioners to receive training in one or more levels of Triple P. To receive the proposed training, practitioners had to agree to participate in the study. Data were collected among trained practitioners through a pre-implementation survey (prior to Triple P training) and a post-implementation survey (1–2 years later). Meanwhile, the practitioners were expected to deliver the various components of Triple P and monitor their Triple P interventions on an ongoing basis, with the support of the research team. This procedure was approved by the relevant ethics research board.

Several means were put in place to ensure optimal implementation of Triple P in the communities. First of all, the implementation was carefully prepared in accordance with the QIF [29]. In particular, the needs and resources of the targeted communities were assessed, as well as their readiness to act in maltreatment prevention. In addition, the differentiation of Triple P from other parenting support programs in use in Quebec was established, in order to ensure possible linkages with other programs. Two local implementation coordinators from each of the communities were hired to act as resource person during all phases of implementation. Their role included mobilizing other partners in the field and acting as a bridge between the research team and the partners.

A local implementation committee was formed in each of the communities, bringing together regional and local partners, i.e., representatives of government authorities (e.g., public health department, youth protection department), the local coordinator for the implementation of the territory, as well as managers or representatives of partner organizations. The mandate of these implementation teams was to plan the concerted implementation of Triple P on their territory.

During the active implementation phase of the program, the local implementation coordinators were mandated to provide supervision, to help the practitioners while promoting their self-regulation, and to help refer parents to the level most suited to their needs. The managers were briefed on their role in supporting practitioners, which included informing the implementation team members of the needs of their staff, providing time and tools to practitioners to become efficient in delivering the program, and working in collaboration with the other organizations to share resources and knowledge. Finally, the research team established procedures to facilitate the work of practitioners, for example, by providing them with an electronic tablet that they could use to show parents intervention materials (Triple P videos and tip sheets, for example) and by encouraging them to document their interventions using specially designed computerized monitoring tools. While the research team was more involved in the planning and coordination of the initiative at the beginning of the project, it took on more of a coaching role over time so that communities partners could take ownership of the initiative and develop their collective capacity for implementation on their own.

### Participants

Participants were 115 practitioners (93% females) trained in at least one level of Triple P in fall 2014 (*n* = 94) or fall 2015 (*n* = 21). Of these, 99 completed the posttest (retention rate: 86%). Participants’ characteristics are presented in Table 1. Posttest completers and non-completers were similar with regard to all sociodemographic variables, except the number of years of experience working with families, with completers having significantly more experience (M = 14.04, SD = 9.41) than non-completers (M = 8.29, SD = 5.33), *t* (26.7) = − 3.35, *p* = .002.

**Table 1:** Sociodemographic Characteristics of Participants (*N* = 99)

	(%)
Academic background	
Early education or specialized education	38.38
Nursing	7.07
Social work or psychology	39.39
Other (administration, non applied social science) or unspecified	15.15
Highest level of education completed	
High school diploma or no diploma	4.04
Technical/academic junior college diploma	32.32
Undergraduate degree	49.49
Post-graduate degree	4.14
Type of organization	
School or child care services	12.12
Non-governmental organizations	22.22
Governmental primary care agencies or child welfare services	65.66
Average number of years of experience working with families	M (SD) 14.04 (9.41)
Total duration of involvement in the initiative (in months)	19.5 (6.8)

### Measures

Variables were assessed using four validated questionnaires completed at pretest and posttest. All measures were translated into French by the research team (except for the PCSC measure that was translated by Triple P International) and contextualized to the implementation of Triple P when applicable. Since the members of the research team are bilingual, including both people whose primary language is French or English, the translation of the questionnaires was the result of collaborative and iterative work between them. The back translation process recommended by some authors [51, 52] was not considered necessary in this context. Internal consistency was calculated for each questionnaire translated and used in the present study to ensure the validity of the measures. A sociodemographic questionnaire was included to collect background information on participants (sex, academic background, discipline, years of experience working with families and type of organization). All translated versions of the questionnaires used in this study are provided as supplementary files (see Additional file 1 for pretest questionnaires and Additional file 2 for posttest questionnaires).

#### Attitudes towards EBPs

Participants’ attitudes towards EBPs were assessed using the Evidence-Based Practice Attitude Scale (EBPAS) [15]. This questionnaire comprises 15 items rated on a Likert-type scale (1 = not at all to 5 = to a very great extent) and divided into four subscales: Appeal (extent to which EBPs are intuitively appealing to the practitioner); Requirements (extent to which the practitioner would adopt an EBP if his/her supervisor required it); Openness (general receptivity to new practices); and Divergence (perceived divergence between EBPs and the current practice). With the exception of the Divergence subscale, higher scores indicate more favorable attitudes towards EBPs. Internal consistency was satisfactory in both Aarons’ [15] original validation study (Chronbach’s alphas for subscales = .80, .90, .78, .59, respectively) and the present study (.73, .93, .87 and .71).

#### Perceived training needs

This variable was assessed using the Training Needs subscale of the Organizational Readiness for Change measure (ORC) [50], comprising 8 items rated on a Likert-type scale (10 = strongly disagree to 50 = strongly agree). In the present study, the last item, relating to “using computerized client assessments,” was removed because it did not apply to the context. The remaining items assessed, for example, the extent to which practitioners felt they needed more training to increase client participation in treatment, monitor client progress or improve client thinking and problem-solving skills. This subscale, conceptualized as a measure of motivational readiness for change, demonstrated good internal consistency in both Lehman et al.’s study [53] (Chronbach’s α = .84) and the present study (*a* =.87).

#### Self-efficacy

The Parent Consultation Skills Checklist (PCSC) [5], translated into French by Triple P International, was used to assess the practitioners’ level of confidence in their skills for working with parents reporting difficulties with their children. This measure, developed by Turner and Sanders [54], is specifically tailored to levels 2, 3, 4, and 5 of the Triple P program. Items refer to both content self-efficacy (e.g., teaching positive parenting principles to parents) and process self-efficacy (e.g., installing and using the audiovisual equipment required for the session) [26], and are rated on a Likert-type scale (1 = not at all confident” to 7 = very confident). This instrument showed good internal consistency in both Turner et al. study’s [5] (Chronbach’s α = .96 to .97 for the difference program levels) and in the present study (Chronbach’s α = .92, .96, .94 and .95, respectively). At pretest, the PCSC was completed just before training in each level of Triple P. When practitioners were trained in more than one level, only the score on their first completed pretest PCSC was used in the analyses. At posttest, practitioners completed a PCSC for each level of Triple P in which they had received training. A mean score for all the completed posttest PCSCs (ranging from 1 to 7) was computed and used in the analyses.

#### Perceived organizational capacity at pretest

This variable was assessed by computing an aggregated score for three subscales of the Factors Related to Program Implementation measure (FRPI) [36]: Ideal Agency, Ideal Staff, and Ideal Champion. This procedure was justified given the high correlation found between these three subscales (*r* ranging from .51 to .80). Practitioners rated 24 Likert-type items assessing the extent to which various characteristics of the agency, staff, and supervisor would be a barrier or an asset to the implementation of Triple P (1 = significant barrier to 5 = significant asset). FRPI items cover different agency characteristics (e.g., perceived coherence of Triple P with organizational mandate, perceived quality of program coordination), staff characteristics (e.g., perceived level of motivation and competence, and communication between team members), and supervisor characteristics (e.g., perceived level of motivation, competence, availability and support). The aggregated score showed good internal consistency in the present study (α = .85).

### Procedures

Pretest surveys were completed a few days prior to Triple P training. Posttest surveys were sent to participants and collected in fall 2016, or earlier if the practitioner was going to be leaving the organization for any reason, such as maternity leave, prolonged sick leave or a change of assignment. To increase the response rate, follow-up calls were made to practitioners who did not return their questionnaire within the prescribed period.

### Statistical analyses

Descriptive analyses of variable distributions revealed no problems related to the conditions of use of any of the planned analyses. A negligible amount of missing data was found for each dependent variable (3.5% on average). Consequently, procedures for handling missing data were deemed unnecessary [55]. Analyses were conducted using SPSS and SPSS macro PROCESS [56].

**Fig. 1:**
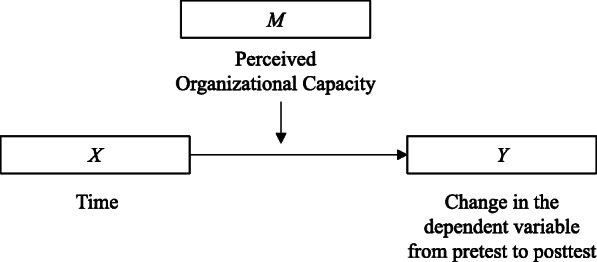
*Illustration of the moderation model tested*. *Note. X* = Predictive variable, *M* = Moderator, *Y* = Predicted variable (i.e. Change in: Appeal of EBPs; Propensity to use an EBP if required by the supervisor; Openness to new practices; Perceived divergence between EBPs and the current practice; Perceived training needs related to working with parents; and Self-efficacy for working with parents)

Using a bootstrapping method, six linear regressions were conducted to test the interaction effect of perceived organizational capacity on the level of change in the dependent variables over time. The six dependent variables were the levels of change in the four attitude subscales of the EBPAS (Appeal, Requirements, Openness and Divergence), the ORC Training Needs subscale and the PCSC Self-efficacy measure; the moderator was the global FRPI score; and the independent variable was time (pretest, posttest). Figure 1 illustrates the moderation model tested.

The Johnson-Neyman procedure, probing interactions with continuous moderators, was performed to determine regions of significance of the interaction effect. This procedure indicates the value of the moderator (i.e., the specific score of the FRPI) at which the interaction effect becomes significant. The advantage of this method is that it provides a more complete picture of the interaction effect and does not require an arbitrary dichotomization of the moderating variable [57].

Regression analyses were conducted to test for the presence of a time effect when the interaction effect was not significant. These analyses controlled for participant characteristics (e.g., level of education, prior experience, type of organization) previously associated in the literature with participants’ attitudes, training needs or self-efficacy [12, 58, 59]. The analyses also controlled for the length of time between pre-test and post-test, since it varied between one and two years depending on participants.

Preliminary analyses involving sociodemographic data showed that only two control variables were significant predictors in some tested models: practitioners’ prior experience working with families (in number of years) and community membership (i.e., working in one health catchment area or the other). Including these variables as covariates did not change the direction, magnitude or significance of the results. More parsimonious models excluding these covariates are thus presented below.

## Results

Average scores for practitioner’s attitudes, perceived training needs and self-efficacy at pretest and posttest are presented in Table 2. The average score for perceived organizational capacity, measured at pretest, was 4.12 (SD = .73).

**Table 2:** Scores for Practitioners’ Attitudes, Perceived Training Needs and Self-Efficacy

Measure	Pretest		Posttest	
	M	SD	M	SD
Appeal	4.22	.56	4.25	.51
Requirements	3.63	.88	3.80	.82
Openness	3.87	.64	3.87	.60
Divergence	2.19	.59	2.32	.57
Perceived training needs	33.75	7.58	29.46	7.49
Self-efficacy	4.73	.93	5.65	.93

*Note. Appeal* = appeal of EBPs, *Requirements* = propensity to use an EBP if required by the supervisor, *Openness* = openness to new practices, *Divergence* = perceived divergence between EBPs and the current practice, *Perceived training needs* = perceived training needs related to working with parents, *Self-efficacy* = self-efficacy for working with parents

As indicated in Table 3, a significant interaction effect of time X perceived organizational capacity was found for Appeal of EBPs, Openness to new practices and Perceived training needs. No significant interaction effect of time X perceived organizational capacity was found for Self-efficacy. However, a main effect of time was observed, with practitioners’ level of self-efficacy for working with parents significantly increasing from pretest to posttest, *F* (1, 201) = 49.83, *p* < .001, *R*^*2*^ = .20, *b* = .87, *t* (201) = 7.06, *p* < .001. The effect size was moderate, with a standardized beta coefficient of .45. No significant interaction or time effects were found for Requirements (propensity to use an EBP if required by the supervisor) or Divergence (perceived divergence between EBPs and the current practice).

**Table 3:** Interaction Effect of Perceived Organizational Capacity on the Level of Change in Practitioners’ Attitudes, Perceived Training Needs and Self-Efficacy, and Time Effect

	*b*	*se*	*β*	*t*	*p*
Interaction effect (time X POC)					
Appeal	−.29	.10	−.39	− 2.87	.005*
Requirements	−.18	.17	−.15	− 1.03	.303
Openness	−.27	.12	−.32	−2.35	.020*
Divergence	.04	.12	.05	.36	.718
Perceived training needs	−4.03	1.62	−.37	−2.48	.014*
Self-efficacy	−.02	.17	−.02	−.14	.890
Time effect					
Requirements	.17	.12	.10	1.44	.153
Divergence	.14	.08	.12	1.75	.082
Self-efficacy	.87	.12	.45	7.06	.000**

*Note. Appeal* = appeal of EBPs, *Requirements* = propensity to use an EBP if required by the supervisor, *Openness* = openness to new practices, Divergence = perceived divergence between EBPs and the current practice, *Perceived training needs* = perceived training needs related to working with parents, *Self-efficacy* = self-efficacy for working with parents. *POC* = perceived organizational capacity. * = *p* < .05, ** = *p* < .001

**Fig. 2:**
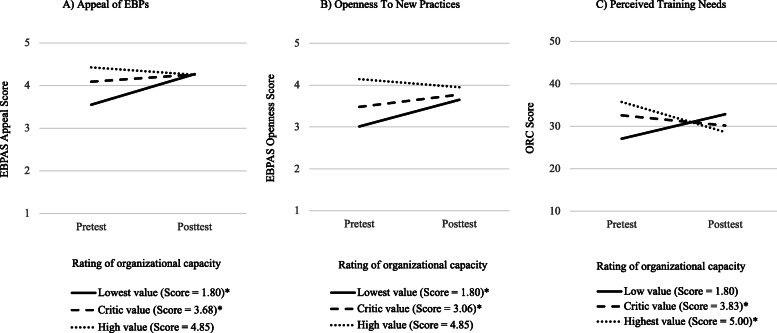
*Moderation of Variables A, B and C Over Time by Perceived Organizational Capacity. Note.* *The moderation effect for this value is in the zone of significance (*p* < .05)

As illustrated in Fig. 2, the magnitude of the positive change in the Appeal of EBPs increased when practitioners’ rating of Organizational capacity was lower at pretest. The Johnson-Newman technique revealed that the interaction effect was significant when the Organizational capacity score was between 1.80 and 3.67, this is, when practitioners tended to perceive more barriers than assets regarding their organization’s capacity to implement Triple P. The effect size varied from large to moderate in this zone of significance, with standardized beta coefficients ranging from 1.35 (FRPI = 1.80) to .32 (FRPI = 3.67). The same pattern was observed for Openness to new practices. Overall, the effect of time on Openness was stronger when practitioners’ rating of Organizational capacity was low. The interaction effect was significant when the FRPI global score was between 1.80 and 3.06 and standardized beta coefficients ranged from 1.03 (FRPI = 1.80) to .47 (FRPI = 3.06). Also, a greater decrease in Perceived training needs was observed when practitioners tended to perceive more assets than barriers regarding their Organization’s capacity to implement Triple P. Specifically, the Johnson-Newman technique revealed that the time X organizational capacity interaction effect was significant when the Organizational capacity score was between 3.83 and 5.00. The effect size varied from moderate to large in this zone of significance, with standardized beta coefficients ranging from −.31 (FRPI = 3.83) to −.91 (FRPI = 5.00).

## Discussion

This study aimed to assess changes over time in the attitudes, perceived training needs and self-efficacy of practitioners involved in the implementation of an EBP, namely, the Triple P program. Results suggest that even before being trained in Triple P, the practitioners as a group showed favorable attitudes towards EBPs, and felt quite confident in their ability to deliver the program components. However, they expressed a moderate need for training related to working with parents. The less confident the practitioners felt regarding their organization’s capacity to implement Triple P at pretest, the greater the extent to which the appeal of EPBs and the practitioners’ openness to new practices increased over the course of implementing this program. Moreover, a higher level of initial confidence regarding their organization’s capacity to implement Triple P was associated with a greater decrease in perceived training needs over time. A moderate increase in self-efficacy over time was seen for all practitioners, regardless of their initial perception of organizational capacity.

These favorable changes in the practitioners’ attitudes, perceived training needs and self-efficacy could reflect the considerable effort made by the local coalitions to ensure a high-quality implementation process [28, 57]. In support of this idea, this study appears to be among the only ones to find positive changes over time in practitioners’ attitudes and training needs towards EBPs. Contrary to the present study, no structured implementation strategy was put in place to support practitioners following their EBP training in the other studies [22, 23]. The authors had suggested that this may be a reason why there was no change in practitioners’ perceptions over time. Moreover, no decrease in attitudes or self-efficacy and no increase in perceived training needs were reported. These findings partially support this study’s hypothesis that positive changes in all variables would be observed, based on the assumption of the QIF that every cycle of implementation would foster learning and improvements that could later be used to start a new cycle [29]. However, no changes were observed in two variables of attitudes, and some of these positive changes were moderated by the practitioners’ initial perception of their organization’s capacity to implement Triple P.

Regarding attitudes towards EBPs, the results indicate that the levels of change in the appeal of EBPs and practitioners’ openness to new practices were moderated by organizational capacity, but not in the direction expected. Indeed, it was hypothesized that a more favorable perception of organizational capacity would predict greater improvements in attitudes, based on previous studies reporting positive associations between attitudes and organizational factors [38, 39]. Instead, this study showed that perceiving more barriers than assets to the implementation of Triple P predicted a greater improvement in the attitudes of practitioners. These findings bring out nuances regarding the suggestion emerging from the implementation literature that initial staff buy-in should be obtained before engaging in any implementation process [15, 57]. For instance, having found that school counselors were more likely to ensure better implementation outcomes when they met initial characteristics such as not being cynical and not being limited by excessive managerial control, Lochman and al [34]. emphasized the need to carefully screen for the staff to be trained before beginning the implementation of a program. While it is likely that minimal staff buy-in at the outset of the implementation of an EBP is necessary in order for the program to be offered, the results of the present study show that such buy-in may not need to be very high or consistent among practitioners. Indeed, in the present study, an initially critical or neutral stance regarding the organization’s capacity to implement an EBP was associated with greater positive changes in both the appeal of EBPs and the practitioners’ openness to new practices over time. As demonstrated by Leathers et al. [23], such an improvement in attitudes could lead to higher engagement in support activities following training (e.g., seeking consultation with a mentor), which could in turn improve implementation and program outcomes.

On the other hand, the results pertaining to training needs confirmed the initial hypothesis of this study, with perceived training needs decreasing over time, especially among practitioners who initially displayed more optimism regarding their organization’s capacity to implement Triple P. It is possible that, having greater confidence in the successful implementation of the program, these practitioners were able to engage more actively in the training and subsequent clinical supervision provided. They may thus have drawn greater benefit from their participation in the implementation process, as reflected in a decrease in their perceived training needs.

In this study, practitioners’ confidence in their skills for delivering Triple P interventions increased moderately over time. The extent of this change was not moderated by perceived organizational capacity. Many studies have shown that active training, such as that for Triple P, tends to increase practitioners’ self-efficacy, an effect that can be maintained over time [5, 60]. The finding of the present study raises the following question: Did the increase observed after two years simply reflect the sustained effect of the initial training or, as hypothesized, could it also have been due to the practitioners’ experience of being involved in a structured and supportive implementation process? Given that the practitioners’ initial appraisal of organizational capacity did not moderate changes in their level of self-efficacy over time, it is possible that the observed changes in self-efficacy were moderated or mediated by other factors which came into play during or after the initial training, such as increased practice with the program or higher perceived benefits for parents. Turner et al. [5] observed, for example, that post-training self-efficacy predicted the level of program use six months later. Without testing their hypothesis, these authors proposed that, in turn, successful use of the program with clients would likely increase practitioners’ level of confidence in their skills and motivate them to use the program again, leading to a positive feedback cycle between self-efficacy and level of use. Moreover, Shapiro, Prinz and Sanders [21] highlighted the role of “early wins” (i.e., early experiences of success with the program and related positive impact on families) on the later experience of providers who reported sustained use of the program over a number of years. It is possible that experiencing such “early wins” builds practitioners’ self-efficacy, assuming that practitioners’ skills for delivering the program, using an effective balance of flexibility and fidelity [61], actually begin to improve [5, 20]. The fact that practitioner’s self-efficacy improved over time independantly of their perception of the organizational capacity could also mean that changes in self-efficacy are more linked to individual factors than contextual and organizational factors. Indeed, Mazzucchelli and Ralph [22] conceptualize self-efficacy as a component of self-regulatory skills, which tend to vary from one practitioner to another even when working in a similar context. It should be noted, however, that these authors do not overlook the influence of external factors, with particular emphasis on how the self-regulatory skills of stakeholders can be increased by different interventions. For example, they recommend that practitioners receive an EBP training with a trainer that help them to self-evaluate and self-reinforce, that they receive peer supervision once they use the EBP and that they be able to monitor their results with their clients. All those elements were generally put in place by the local coalitions in the current implementation initiative, and therefore could have helped improve self-efficacy in practitioners over time, as seen in this study’s results.

Regardless of the reason why these positive changes in self-efficacy occurred, these results are of clinical importance, since higher self-efficacy is associated with better actual skills in performing the task and greater resilience to stressors [18, 19]. Therefore, a higher mastery of the skills needed to deliver the program components and greater persistence through the numerous challenges of implementation should lead to better outcomes for families [19, 21].

This study has some limitations. First, the absence of a comparison group makes it impossible to determine whether the changes observed among the practitioners over time were actually due to their participation in the implementation process, or to other factors such as the simple passage of time or mere exposure to Triple P training. An experimental design including randomization of practitioners in experimental and control groups would also have eliminated the threat of statistical regression to the mean that can occur in a pretest/posttest study design [62]. Second, the results found for attitudes towards EBPs were limited by the instrument chosen. For instance, the possibility that the perceived divergence decreased with regard to Triple P specifically could not be captured by the instrument used, since the EBPAS items focus on manual-based programs in general [14, 15, 63]. Third, the implementation portrait provided in this study may predominantly reflect the views of more experienced practitioners, since less experienced practitioners were underrepresented in the sample. However, the fact that the results were not affected by the amount of previous experience when this control variable was included in the analyses raises confidence that this limit does not represent a threat to the validity of the present findings. Fourth, the French translations of the questionnaires used in this study were not subjected to a full adaptation process in accordance with the guidelines of the International Test Commission (ITC) [64]. These guidelines recommend in particular that the translated version of the instrument be tested initially to ensure its validity with the new population. In this study, the sole analyses conducted on the translated questionnaires were aimed at verifying their internal consistency.

## Conclusion

At a time when the scientific community and decision-makers are promoting the use of practices based on the best available evidence, it is interesting to see that the practitioners who participated in the present study generally displayed confidence and enthusiasm at the beginning of the implementation of this EBP. Is it even more encouraging to observe that being involved in this process appears to have positively influenced the perceptions of practitioners who were less confident at the outset. These findings support the idea that the efforts invested in the implementation process of EBPs by communities wishing to adopt such programs are worthwhile, even when some staff members initially appear to be less inclined to engage in the initiative.

## Supplementary Information

**Additional file 1.** Questionnaire de pré-implantation/Pretest questionnaire, *Description of data: Set of questionnaires completed by participants at pretest, including a sociodemographic questionnaire and the French translation of the questionnaires EBPAS, ORC and FRPI. The French translation of the questionnaire PCSC was provided to participants by Triple P International before the Triple P training sessions and is therefore not included in Additional file 1. It is however included in Additional file 2.

**Additional file 2.** Questionnaire de fin de participation/Postest questionnaire, *Description of data: Set of questionnaires completed by participants at posttest, including the French translation of the questionnaires EBPAS, ORC, FRPI and PCSC.

## Data Availability

The data that support the findings of this study are available from Prof. Marie-Hélène Gagné but restrictions apply to the availability of these data, which were used under license for the current study, and so are not publicly available. Data are however available from the authors upon reasonable request and with permission of Prof. Marie-Hélène Gagné, who can be reached by e-mail: marie-helene.gagne@psy.ulaval.ca.

## Abbreviations

EBP: Evidence-based program
EBPAS: Evidence-Based Practice Attitude Scale
FRPI: Factors Related to Program Implementation
ORC: Organizational Readiness for Change
PCSC: Parent Consultation Skills Checklist
Triple P: Positive Parenting Program
QIF: Quality Implementation Framework
