# Supplementary material for: Changes in practitioners’ attitudes, perceived training needs and self-efficacy over the implementation process of an evidence-based parenting program
Source: BMC Health Serv Res. 2020 Nov 27;20:1092. doi: 10.1186/s12913-020-05939-3 (PMC7694945; doi:10.1186/s12913-020-05939-3)
Supplement: Supplementary file 1 — Additional file 1. Questionnaire de pré-implantation/Pretest questionnaire, *Description of data: Set of questionnaires completed by participants at pretest, including a sociodemographic questionnaire and the French translation of the questionnaires EBPAS, ORC and FRPI. The French translation of the questionnaire PCSC was provided to participants by Triple P International before the Triple P training sessions and is therefore not included in Additional file 1. It is however included in Additional file 2. [file 12913_2020_5939_MOESM1_ESM.pdf]

Mise en œuvre de *Triple P à Québec*

Questionnaire pré-implantation

Pour les intervenants

Vous êtes sollicité-e pour répondre à ce questionnaire puisque vous serez bientôt formé-e pour expérimenter le programme *Triple P* sur le territoire du CLSC Orléans.

Ce questionnaire porte sur vos perceptions face à l'utilisation d'un programme à données probantes et sur des composantes du contexte organisationnel et de la collaboration entourant la mise en œuvre de *Triple P.*

Il comprend 5 sections et totalise 76 questions. Il prend environ 30 minutes à remplir. Merci de prendre le temps d'y répondre.

Il n'y a pas de bonnes ou de mauvaises réponses aux questions. Les réponses réfléchies et honnêtes seront les plus utiles. ***Veuillez répondre aux questions en sélectionnant <u>un seul choix de réponse</u> par question.***

Consignes pour le retour du questionnaire

Veuillez SVP rapporter ce questionnaire rempli, placé dans l'enveloppe vierge ci-jointe, lors de votre formation Triple P. Il sera récupéré avant le début de la formation. Merci!

Réservé à l'équipe de recherche :

Code : ________________ **Date de réception :** ____________________

Données sociodémographiques

Veuillez cocher les caractéristiques qui correspondent à votre profil.

1. **Sexe :**

- ☐ Homme
- ☐ Femme

2. **Dernier diplôme obtenu :**

- ☐ Diplôme d'études professionnelles (DEP)
- ☐ Diplôme d'études collégiales (DEC)
- ☐ Baccalauréat
- ☐ Maîtrise ou doctorat

3. **Domaine / discipline de formation :**

- ☐ Travail social
- ☐ Psychoéducation
- ☐ Éducation spécialisée
- ☐ Éducation à l'enfance
- ☐ Enseignement
- ☐ Psychologie
- ☐ Autre : ______________________________

4. **Nombre d'années d'expérience dans ce domaine : ___________**

5. **Type d'organisation dans lequel vous travaillez actuellement :**

- ☐ Établissement du réseau de la santé et des services sociaux (CSSS, CLSC, CJ)
- ☐ Organisme communautaire
- ☐ Milieu de garde (centre de la petite enfance ou milieu de garde familial)
- ☐ Milieu de l'éducation (commission scolaire, école)
- ☐ Milieu municipal (ville, arrondissement, loisir, bibliothèque)
- ☐ Autre : ______________________________

6. **Nombre d'années d'expérience dans cette organisation : ___________**

SECTION A
Attitudes des intervenant-e-s
Evidence-based Practice Attitude Scale (Aarons, 2004)

Les questions suivantes portent sur vos sentiments au sujet de l'utilisation de nouveaux types de programmes ou d'interventions. Indiquez dans quelle mesure vous êtes d'accord avec chacun des énoncés suivants :

		Pas du tout	Dans une faible mesure	Dans une certaine mesure	Dans une large mesure	Dans une très large mesure
1	J'aime utiliser de nouveaux types de programmes / interventions pour aider mes clients.					
2	Je suis prêt-e à essayer de nouveaux types de programmes / interventions, même si je dois suivre un manuel d'intervention.					
3	Je sais mieux que des chercheurs universitaires comment m'occuper de mes clients.					
4	Je suis prêt à utiliser des types de programmes / interventions nouveaux et différents, développés par des chercheurs.					
5	Les programmes/ interventions basés sur la recherche ne sont pas utiles sur le plan clinique.					
6	L'expérience clinique est plus importante que l'utilisation de programmes/ interventions manualisés*.					
7	Je n'utiliserais pas de programmes/ interventions manualisés*.					
8	J'essayerais un nouveau programme/intervention même si c'était très différent de ce que j'ai l'habitude de faire.					

** Les programmes ou les interventions « manualisés » réfèrent à toute intervention dont les directives ou les composantes spécifiques sont décrites dans un manuel, ou qui doivent être suivies de façon structurée ou prédéterminée.*

**Pour les questions 9 à 15 : Si vous receviez une formation pour un programme / intervention qui est nouveau pour vous, quelle serait la probabilité que vous l'adoptiez :**		Pas du tout	Dans une faible mesure	Dans une certaine mesure	Dans une large mesure	Dans une très large mesure
9	… s'il est intuitivement attrayant?					
10	… s'il « fait du sens » pour vous?					
11	… s'il est exigé par votre supérieur immédiat?					
12	… s'il est exigé par votre établissement/organisme?					
13	… s'il est exigé par le ministère?					
14	… s'il a été utilisé par des collègues qui en sont satisfaits?					
15	… si vous sentez que vous avez eu suffisamment de formation pour l'utiliser correctement?					

SECTION B
Le degré de préparation de l'organisation à offrir Triple P
Organisation Readiness for Change (Lehman, Greener et Simpson, 2002)

Implanter un nouveau programme d'intervention (en l'occurrence, Triple P) est exigeant pour une organisation. Les questions suivantes visent à évaluer le degré de préparation de votre établissement ou organisme à faire face à cette situation.

Pour les prochaines questions, pensez à vos installations et à vos locaux *en lien avec l'implantation de Triple P.* Jusqu'à quel point êtes-vous en accord ou en désaccord avec chacun des énoncés suivants?

		Fortement en désaccord	En désaccord	Incertain	En accord	Fortement en accord	Ne s' applique
1	Vos bureaux et vos équipements sont adéquats.						
2	Vos installations sont adéquates pour y mener des interventions de groupe.						
3	Vos bureaux permettent l'intimité et la confidentialité requises pour les interventions individuelles.						
4	Vos installations fournissent aux parents un espace confortable d'accueil/attente.						

Pour les prochaines questions, <u>pensez à l'équipe</u> ou à <u>l'organisme</u> dont vous faite partie. Jusqu'à quel point êtes-vous en accord ou en désaccord avec chacun des énoncés suivants?

		Fortement en désaccord	En désaccord	Incertain	En accord	Fortement en accord	Ne s' applique
5	L'équipe/organisme compte suffisamment d'intervenants pour répondre aux besoins actuels des parents.						
6	Une équipe d'intervention plus grande est requise pour rencontrer les exigences du programme Triple P.						
7	Le roulement de personnel est un problème pour le programme Triple P.						
8	Les intervenants de votre équipe/organisme sont en mesure de passer suffisamment de temps avec les parents.						
9	Votre équipe d'intervention possède les habiletés dont elle a besoin pour faire son travail.						
10	Le personnel clinique est bien formé.						

Pour les prochaines questions, <u>pensez à votre propre travail avec des parents</u>. Diriez-vous que vous avez besoin de plus de formation pour :

		Fortement en désaccord	En désaccord	Incertain	En accord	Fortement en accord	Ne s' applique
11	Évaluer les problèmes et les besoins des parents.						
12	Augmenter la participation des parents à l'intervention.						
13	Suivre la progression des parents.						
14	Améliorer votre relation avec les parents.						
15	Améliorer les habiletés de réflexion et de résolution de problème des parents.						
16	Améliorer la gestion des comportements chez les parents.						
17	Améliorer l'attention des parents lors d'une intervention de groupe.						

SECTION C
La collaboration autour de Triple P
Integration of Human Services Measure (Browne, 2002)

Dans le tableau présenté à la page suivante, vous trouverez la liste des établissements et organismes qui participent à la mise en œuvre de Triple P sur le territoire du CLSC Orléans.

Pour chaque établissement/organisme listé, vous devez évaluer le niveau de collaboration que vous entretenez avec cet établissement/organisme.

(1) La partie gauche du tableau permet d'indiquer le niveau de collaboration actuel
(2) La partie droite du tableau permet d'indiquer le niveau de collaboration souhaité

Inscrivez vos réponses (0 à 4) dans les cases grisées. N'inscrivez rien vis-à-vis de votre propre établissement/organisme – laissez l'espace vide.

Attention : l'échelle de réponse de 0 à 4 indique un niveau croissant d'intensité dans la collaboration entre les organisations. En d'autres mots, le niveau 1 est préalable au niveau 2 puisque pour communiquer il est nécessaire de d'abord être au courant de l'existence de l'autre. De la même manière, le niveau 2 est préalable au niveau 3, car pour coopérer, il faut d'abord communiquer. Merci d'inscrire vos réponses en tenant compte de cette précision.

<table>
<tr><th colspan="2">Jusqu’à quel point
ÊTES-VOUS
impliqué-e avec les organisations suivantes?</th><th rowspan="2">**Établissements et organismes**</th><th colspan="2">Jusqu’à quel point
DEVRIEZ-VOUS ÊTRE
impliqué-e avec les organisations suivantes?</th></tr>
<tr><th>**Échelle**</th><th>**Cote** (0-4)</th><th>**Cote** (0-4)</th><th>**Échelle**</th></tr>
<tr><td rowspan="12">**0 = Aucune connaissance**
Je ne connais pas cet organisme

1 = Connaissance
je connais cet organisme mais ne réalise aucune activité conjointe avec celui-ci.

2 = Communication
Je communique activement et échange de l’information avec cet organisme

3 = Coopération
Moi et des membres de cet organisme utilisons réciproquement notre connaissance de l’autre pour guider et modifier la planification de nos activités en vue d’améliorer les liens entre nos services

4 = Collaboration
Moi et des membres de cet organisme **planifions conjointement** nos offres de services et pouvons modifier nos activités sur la base de l’avis ou de la contribution de l’autre.</td><td></td><td>CSSS Québec-Nord (CLSC Orléans)</td><td></td><td rowspan="12">**0 = Aucune connaissance**
Je n’ai pas à connaître cet organisme

1 = Connaissance
Je devrais connaître cet organisme même si aucune activité conjointe n’est réalisée avec celui-ci.

2 = Communication
Je devrais communiquer activement et échanger de l’information avec cet organisme

3 = Coopération
Moi et des membres de cet organisme devrions réciproquement utiliser notre connaissance de l’autre pour guider et modifier la planification de nos activités en vue d’améliorer les liens entre nos services

4 = Collaboration
Moi et des membres de cet organisme **devrions planifier conjointement** nos offres de services et accepter de modifier nos activités sur la base de l’avis ou de la contribution de l’autre.</td></tr>
<tr><td></td><td>Centre Jeunesse de Québec – institut universitaire</td><td></td></tr>
<tr><td></td><td>Commission scolaire des Premières-Seigneuries (ou ses écoles)</td><td></td></tr>
<tr><td></td><td>Matinée Frimousse</td><td></td></tr>
<tr><td></td><td>Re-Fa-Vie</td><td></td></tr>
<tr><td></td><td>Ressources familiales Côte-de-Beaupré</td><td></td></tr>
<tr><td></td><td>CPE Percée de soleil</td><td></td></tr>
<tr><td></td><td>CPE L’Enchanté</td><td></td></tr>
<tr><td></td><td>CPE L’éveil Enfantin</td><td></td></tr>
<tr><td></td><td>CPE La Petite école de Beauport</td><td></td></tr>
<tr><td></td><td>CPE Hippo-Plus</td><td></td></tr>
<tr><td></td><td>CPE La Ritournelle</td><td></td></tr>
</table>

SECTION D
Les facteurs susceptibles d'aider ou de nuire à la mise en œuvre de Triple P
FRPI (Mihalic et Irwin, 2003)

Les questions suivantes visent à identifier les facteurs susceptibles d'aider, ou de nuire, à la mise en œuvre de Triple P dans votre établissement / organisme.

Caractéristiques de l'établissement / organisme

	**<u>Question</u> : En prévision de la mise en œuvre de Triple P, dans quelle mesure chacun des facteurs suivants sera-t-il un atout ou un obstacle, selon vous?**	Obstacle important	Obstacle modéré	Ni un atout, ni un obstacle	Atout modéré	Atout important
1	La participation du personnel clé dans la planification, la prise de décision et la résolution de problème en ce qui concerne Triple P.					
2	Le soutien administratif et le leadership (soutien moral) en lien avec Triple P.					
3	Les voies de communication ouvertes entre la direction de l'établissement/organisme, la coordonnatrice locale d'implantation et les intervenants, en ce qui concerne Triple P.					
4	La cohérence entre Triple P et les autres programmes et objectifs de l'établissement/organisme.					
5	La cohésion et collaboration entre tous les acteurs impliqués dans l'offre du programme Triple P.					
6	La clarté des objectifs et des procédures de Triple P.					
7	La clarté de la structure hiérarchique en ce qui concerne Triple P.					
8	La stabilité du personnel (taux de roulement).					
9	La présence d'un coordonnateur de programme, ou d'un « champion » pour Triple P.					

**<u>Question</u> : En prévision de la mise en œuvre de Triple P, dans quelle mesure chacun des facteurs suivants sera-t-il un atout ou un obstacle, selon vous?**		Obstacle important	Obstacle modéré	Ni un atout, ni un obstacle	Atout modéré	Atout important
10	Les installations de l'établissement/organisme en fonction de l'offre de Triple P.					
11	Le soutien financier pour Triple P.					
12	L'allocation de ressources suffisantes pour Triple P.					
13	Le climat politique qui entoure l'offre de Triple P.					

Caractéristiques des intervenant-e-s appelé-e-s à offrir Triple P

**<u>Question</u> : En prévision de la mise en œuvre de Triple P, dans quelle mesure chacun des facteurs suivants sera-t-il un atout ou un obstacle, selon vous?**		Obstacle important	Obstacle modéré	Ni un atout, ni un obstacle	Atout modéré	Atout important
14	L'adhésion / soutien des intervenants vis-à-vis de Triple P.					
15	La motivation des intervenants vis-à-vis de Triple P.					
16	Les habiletés et les connaissances des intervenants à propos de Triple P (qualité de l'intervention).					
17	Les intervenants ont du temps à consacrer à la mise en œuvre de Triple P.					
18	Les intervenants accordent la priorité au programme Triple P.					
19	Le bassin d'intervenants disponibles à l'embauche.					
20	La communication avec d'autres intervenants, équipes et gestionnaires qui offrent Triple P.					

Caractéristiques du chef d'équipe*

** L'expression « chef d'équipe » a été choisie pour désigner la personne qui gère ou supervise l'équipe d'intervention, ou le supérieur immédiat des intervenant-e-s affecté-e-s à l'offre du programme Triple P.*

**Question : En prévision de la mise en œuvre de Triple P, dans quelle mesure chacun des facteurs suivants sera-t-il un atout ou un obstacle, selon vous?**		Obstacle important	Obstacle modéré	Ni un atout, ni un obstacle	Atout modéré	Atout important
21	L'adhésion / soutien du chef d'équipe vis-à-vis de Triple P.					
22	La motivation du chef d'équipe vis-à-vis de Triple P.					
23	Les habiletés et les connaissances du chef d'équipe à propos de Triple P (qualité de la supervision).					
24	Le temps accordé par le chef d'équipe à la coordination de Triple P.					

Caractéristiques des formations Triple P

**Question : En prévision de la mise en œuvre de Triple P, dans quelle mesure chacun des facteurs suivants sera-t-il un atout ou un obstacle, selon vous?**		Obstacle important	Obstacle modéré	Ni un atout, ni un obstacle	Atout modéré	Atout important
25	La disponibilité des formations Triple P.					
26	La qualité des formations Triple P.					

Merci de votre collaboration !

Et n'oubliez pas d'apporter votre questionnaire rempli lors de votre formation Triple P !
