# Supplementary material for: Changes in practitioners’ attitudes, perceived training needs and self-efficacy over the implementation process of an evidence-based parenting program
Source: BMC Health Serv Res. 2020 Nov 27;20:1092. doi: 10.1186/s12913-020-05939-3 (PMC7694945; doi:10.1186/s12913-020-05939-3)
Supplement: Supplementary file 2 — Additional file 2. Questionnaire de fin de participation/Postest questionnaire, *Description of data: Set of questionnaires completed by participants at posttest, including the French translation of the questionnaires EBPAS, ORC, FRPI and PCSC. [file 12913_2020_5939_MOESM2_ESM.pdf]

Mise en œuvre de *Triple P à Québec*

Questionnaire de fin de participation

Pour les intervenants

Vous êtes sollicité-e à nouveau pour répondre à ce questionnaire puisque vous avez contribué à l'expérimentation du programme *Triple P* sur le territoire du CLSC Orléans.

Ce questionnaire porte sur diverses composantes du contexte partenarial et organisationnel entourant Triple P, de la collaboration entourant sa mise en œuvre et de vos perceptions face à l'utilisation d'un programme à données probantes*.*

Il comprend uniquement des questions à choix multiples, réparties en six sections, qui vous demanderont entre 30 et 45 minutes à compléter. Merci de prendre le temps d'y répondre.

Il n'y a pas de bonnes ou de mauvaises réponses aux questions. Les réponses réfléchies et honnêtes seront les plus utiles. ***Veuillez répondre en sélectionnant <u>un seul choix de réponse</u> par question.***

Nous vous remercions de votre participation

Pour toute question, veuillez vous adresser à :

Julie Goulet
418-656-2131 poste 3789
julie.goulet@jefar.ulaval.ca
Chaire de partenariat en prévention de la maltraitance

Réservé à l'équipe de recherche :

Code : ______________ **Date de réception :** ________________

Données sociodémographiques

Veuillez cocher les caractéristiques qui correspondent à votre profil.

1. **Sexe :**

- ☐ Homme
- ☐ Femme

2. **Dernier diplôme obtenu :**

- ☐ Diplôme d'études professionnelles (DEP)
- ☐ Diplôme d'études collégiales (DEC)
- ☐ Baccalauréat
- ☐ Maîtrise ou doctorat

3. **Domaine / discipline de formation :**

- ☐ Travail social
- ☐ Psychoéducation
- ☐ Éducation spécialisée
- ☐ Éducation à l'enfance
- ☐ Enseignement
- ☐ Psychologie
- ☐ Autre : ______________________________

4. **Nombre d'années d'expérience dans ce domaine : ___________**

5. **Type d'organisation dans lequel vous travaillez actuellement :**

- ☐ Établissement du réseau de la santé et des services sociaux (CSSS, CLSC, CJ)
- ☐ Organisme communautaire
- ☐ Milieu de garde (centre de la petite enfance ou milieu de garde familial)
- ☐ Milieu de l'éducation (commission scolaire, école)
- ☐ Milieu municipal (ville, arrondissement, loisir, bibliothèque)
- ☐ Autre : ______________________________

6. **Nombre d'années d'expérience dans cette organisation : ___________**

SECTION A
Attitudes des intervenants
Evidence-based Practice Attitude Scale (Aarons, 2004)

Les questions suivantes portent sur vos sentiments au sujet de l'utilisation de nouveaux types de programmes ou d'interventions. Indiquez dans quelle mesure vous êtes d'accord avec chacun des énoncés suivants :

		Pas du tout	Dans une faible mesure	Dans une certaine	Dans une large mesure	Dans une très large mesure
1	J'aime utiliser de nouveaux types de programmes / interventions pour aider mes clients.	0	1	2	3	4
2	Je suis prêt-e à essayer de nouveaux types de programmes / interventions, même si je dois suivre un manuel d'intervention.	0	1	2	3	4
3	Je sais mieux que des chercheurs universitaires comment m'occuper de mes clients.	0	1	2	3	4
4	Je suis prêt-e à utiliser des types de programmes / interventions nouveaux et différents, développés par des chercheurs.	0	1	2	3	4
5	Les programmes/ interventions basés sur la recherche ne sont pas utiles sur le plan clinique.	0	1	2	3	4
6	L'expérience clinique est plus importante que l'utilisation de programmes/ interventions manualisés*.	0	1	2	3	4
7	Je n'utiliserais pas de programmes/ interventions manualisés*.	0	1	2	3	4
8	J'essayerais un nouveau programme/intervention même si c'était très différent de ce que j'ai l'habitude de faire.	0	1	2	3	4

** Les programmes ou les interventions « manualisés » réfèrent à toute intervention dont les directives ou les composantes spécifiques sont décrites dans un manuel, ou qui doivent être suivies de façon structurée ou prédéterminée.*

	**<u>Pour les questions 9 à 15</u> : Si vous avez reçu une formation pour un programme / intervention qui est nouveau pour vous, quelle serait la probabilité que vous l'adoptiez :**	Pas du tout	Dans une faible mesure	Dans une certaine mesure	Dans une large mesure	Dans une très large mesure
9	… s'il est intuitivement attrayant?	0	1	2	3	4
10	… s'il « fait du sens » pour vous?	0	1	2	3	4
11	… s'il est exigé par votre supérieur immédiat?	0	1	2	3	4
12	… s'il est exigé par votre établissement/organisme?	0	1	2	3	4
13	… s'il est exigé par le ministère?	0	1	2	3	4
14	… s'il a été utilisé par des collègues qui en sont satisfaits?	0	1	2	3	4
15	… si vous sentez que vous avez eu suffisamment de formation pour l'utiliser correctement?	0	1	2	3	4

SECTION B
La collaboration entre les établissements et organismes mobilisés autour de Triple P
***Integration of Human Services Measure* (Browne, 2002)**

Dans le tableau présenté à la page suivante, vous trouverez la liste des établissements et organismes qui ont participé à la mise en œuvre de Triple P sur le territoire du CLSC Orléans.

Pour chaque établissement/organisme listé, vous devez évaluer le niveau de collaboration que votre propre organisation entretient avec cet établissement/organisme.

(1) La partie gauche du tableau permet d'indiquer le niveau de collaboration actuel
(2) La partie droite du tableau permet d'indiquer le niveau de collaboration souhaité

Inscrivez vos réponses (0 à 4) dans les cases grisées. N'inscrivez rien vis-à-vis de votre propre établissement/organisme – laissez l'espace vide.

Attention : l'échelle de réponse de 0 à 4 indique un niveau croissant d'intensité dans la collaboration entre les organisations. En d'autres mots, le niveau 1 est préalable au niveau 2 puisque pour communiquer il est nécessaire de d'abord être au courant de l'existence de l'autre. De la même manière, le niveau 2 est préalable au niveau 3, car pour coopérer, il faut d'abord communiquer. Merci d'inscrire vos réponses en tenant compte de cette précision.

<table>
<tr><th colspan="2">Jusqu'à quel point
ÊTES-VOUS
(votre organisation) **impliqué-e** avec les organisations suivantes?</th><th rowspan="2">**Établissements et organismes**</th><th colspan="2">Jusqu'à quel point
DEVRIEZ-VOUS ÊTRE
(votre organisation) **impliqué-e** avec les organisations suivantes?</th></tr>
<tr><th>**Échelle**</th><th>**Cote** (0-4)</th><th>**Cote** (0-4)</th><th>**Échelle**</th></tr>
<tr><td rowspan="13">**0 = Aucune connaissance**
Votre organisation ne connaît pas cet organisme

1 = Connaissance
Votre organisation connaît cet organisme mais ne réalise aucune activité conjointe avec celui-ci.

2 = Communication
Votre organisation communique activement et échange de l'information avec cet organisme

3 = Coopération
Votre organisation et cet organisme utilisent réciproquement leur connaissance de l'autre pour guider et modifier la planification de ses activités en vue d'améliorer les liens entre les services

4 = Collaboration
Votre organisation et cet organisme **planifient conjointement** leur offre de services et peuvent modifier leurs activités sur la base de l'avis ou de la contribution de l'autre.</td><td></td><td>CIUSSS Capitale-Nationale (CLSC Orléans)</td><td></td><td rowspan="13">**0 = Aucune connaissance**
Votre organisation n'a pas à connaître cet organisme

1 = Connaissance
Votre organisation devrait connaître cet organisme même si aucune activité conjointe n'est réalisée avec celui-ci.

2 = Communication
Votre organisation devrait communiquer activement et échanger de l'information avec cet organisme

3 = Coopération
Votre organisation et cet organisme devraient réciproquement utiliser leur connaissance de l'autre pour guider et modifier la planification de ses activités en vue d'améliorer les liens entre les services

4 = Collaboration
Votre organisation et cet organisme **devraient planifier conjointement** leur offre de services et accepter de modifier leurs activités sur la base de l'avis ou de la contribution de l'autre.</td></tr>
<tr><td></td><td>CIUSSS Capitale-Nationale (Centre jeunesse de Québec)</td><td></td></tr>
<tr><td></td><td>Commission scolaire des Premières-Seigneuries</td><td></td></tr>
<tr><td></td><td>École de la Pléiade</td><td></td></tr>
<tr><td></td><td>École de l'Harmonie</td><td></td></tr>
<tr><td></td><td>École de l'Ile d'Orléans</td><td></td></tr>
<tr><td></td><td>École de la Châtelaine et de la Place-de-l'Éveil</td><td></td></tr>
<tr><td></td><td>Matinée Frimousses</td><td></td></tr>
<tr><td></td><td>Re-Fa-Vie</td><td></td></tr>
<tr><td></td><td>Ressources familiales Côte-de-Beaupré</td><td></td></tr>
<tr><td></td><td>CPE L'Enchanté</td><td></td></tr>
<tr><td></td><td>CPE La petite école de Beauport</td><td></td></tr>
<tr><td></td><td>CPE L'éveil Enfantin (désormais CPE Seigneuriale)</td><td></td></tr>
</table>

SECTION C
Le degré de préparation de l'organisation à offrir Triple P
Organizational readiness for change **(Lehman, Greener et Simpson, 2002)**

Implanter un nouveau programme d'intervention (en l'occurrence, Triple P) est exigeant pour une organisation. Les questions suivantes visent à évaluer comment votre établissement ou organisme a fait face à cette situation.

	Pour les prochaines questions, pensez à vos **installations** et à vos **locaux** <u>en lien avec l'implantation de Triple P</u>. ***Jusqu'à quel point êtes-vous en accord ou en désaccord avec chacun des énoncés suivants?***	Fortement en désaccord	En désaccord	Incertain	En accord	Fortement en accord	Ne s' applique pas
1	Vos bureaux et vos équipements sont adéquats.						
2	Vos installations sont adéquates pour y mener des interventions de groupe.						
3	Vos bureaux permettent l'intimité et la confidentialité requises pour les interventions individuelles.						
4	Vos installations fournissent aux parents un espace confortable d'accueil/attente.						

	Pour les prochaines questions, pensez à **l'équipe** ou à **l'organisme** <u>dont vous faites partie</u>. ***Jusqu'à quel point êtes-vous en accord ou en désaccord avec chacun des énoncés suivants?***	Fortement en désaccord	En désaccord	Incertain	En accord	Fortement en accord	Ne s' applique pas
5	Vous avez suffisamment d'intervenants pour répondre aux besoins actuels des parents.						
6	Une équipe d'intervention plus grande est requise pour rencontrer les exigences du programme Triple P.						
7	Le roulement de personnel est un problème pour le programme Triple P.						
8	Vos intervenants sont en mesure de passer suffisamment de temps avec les parents.						
9	Votre équipe d'intervention possède les habiletés dont elle a besoin pour faire son travail.						
10	Votre personnel clinique est bien formé.						

Pour les prochaines questions, pensez à **votre propre travail avec des parents**. ***Diriez-vous que vous avez besoin de plus de formation pour :***		Fortement en désaccord	En désaccord	Incertain	En accord	Fortement en accord	Ne s' applique pas
11	Évaluer les problèmes et les besoins des parents.						
12	Augmenter la participation des parents à l'intervention.						
13	Suivre la progression des parents.						
14	Améliorer votre relation avec les parents.						
15	Améliorer les habiletés de réflexion et de résolution de problème des parents.						
16	Améliorer la gestion des comportements chez les parents.						
17	Améliorer l'attention des parents lors d'une intervention de groupe.						

SECTION D
Les facteurs susceptibles d'aider ou de nuire à la mise en œuvre de Triple P
***FRPI* (Mihalic et Irwin, 2003)**

Les questions suivantes visent à identifier les facteurs susceptibles d'avoir aidé, ou d'avoir nui, à la mise en œuvre de Triple P dans votre établissement / organisme.

Caractéristiques de l'établissement / organisme

	**<u>Question</u> : En pensant à la mise en œuvre de Triple P, dans quelle mesure chacun des facteurs suivants a-t-il été un atout ou un obstacle, selon vous?**	Obstacle important	Obstacle modéré	Ni un atout, ni un obstacle	Atout modéré	Atout important
1	La participation du personnel clé dans la planification, la prise de décision et la résolution de problème en ce qui concerne Triple P					
2	Le soutien administratif et le leadership (soutien moral) en lien avec Triple P					
3	Les voies de communication ouvertes entre la direction de l'établissement/organisme, la coordonnatrice locale d'implantation et les intervenants, en ce qui concerne Triple P					
4	La cohérence entre Triple P et les autres programmes et objectifs de l'établissement/organisme					
5	La cohésion et collaboration entre tous les acteurs impliqués dans l'offre du programme Triple P					
6	La clarté des objectifs et des procédures de Triple P					
7	La clarté de la structure hiérarchique en ce qui concerne Triple P					
8	La stabilité du personnel (taux de roulement)					
9	La présence d'un coordonnateur de programme, ou d'un « champion » pour Triple P					

	**Question : En pensant à la mise en œuvre de Triple P, dans quelle mesure chacun des facteurs suivants a-t-il été un atout ou un obstacle, selon vous?**	Obstacle important	Obstacle modéré	Ni un atout, ni un obstacle	Atout modéré	Atout important
10	Les installations de l'établissement/organisme en fonction de l'offre de Triple P					
11	Le soutien financier pour Triple P					
12	L'allocation de ressources suffisantes pour Triple P					
13	Le climat politique qui entourait l'offre de Triple P					

Caractéristiques des intervenant-e-s offrant Triple P

	**Question : En pensant à la mise en œuvre de Triple P, dans quelle mesure chacun des facteurs suivants a-t-il été un atout ou un obstacle, selon vous?**	Obstacle important	Obstacle modéré	Ni un atout, ni un obstacle	Atout modéré	Atout important
14	L'adhésion / soutien des intervenants vis-à-vis de Triple P					
15	La motivation des intervenants vis-à-vis de Triple P					
16	Les habiletés et les connaissances des intervenants à propos de Triple P (qualité de l'intervention)					
17	Les intervenants ont eu du temps à consacrer à la mise en œuvre de Triple P					
18	Les intervenants ont accordé la priorité au programme Triple P					
19	Le bassin d'intervenants disponibles à l'embauche					
20	La communication avec d'autres intervenants, équipes et gestionnaires qui offrent Triple P					

Caractéristiques du chef d'équipe*

** L'expression « chef d'équipe » a été choisie pour désigner la personne qui gère ou supervise l'équipe d'intervention, ou le supérieur immédiat des intervenants affectés à l'offre du programme Triple P.*

**<u>Question</u> : En pensant à la mise en œuvre de Triple P, dans quelle mesure chacun des facteurs suivants a-t-il été un atout ou un obstacle, selon vous?**		Obstacle important	Obstacle modéré	Ni un atout, ni un obstacle	Atout modéré	Atout important
21	L'adhésion / soutien du chef d'équipe vis-à-vis de Triple P					
22	La motivation du chef d'équipe vis-à-vis de Triple P					
23	Les habiletés et les connaissances du chef d'équipe à propos de Triple P (qualité de la supervision)					
24	Le temps accordé par le chef d'équipe à la coordination de Triple P					

Caractéristiques des formations Triple P

**<u>Question</u> : En pensant à la mise en œuvre de Triple P, dans quelle mesure chacun des facteurs suivants a-t-il été un atout ou un obstacle, selon vous?**		Obstacle important	Obstacle modéré	Ni un atout, ni un obstacle	Atout modéré	Atout important
25	La disponibilité des formations Triple P					
26	La qualité des formations Triple P					

SECTION E
Utilisation de Triple P et développement des compétences
Incluant le PCSC **(Turner et al., 2011)**

Les prochaines questions visent à recueillir des informations sur votre utilisation de Triple P, ainsi que votre point de vue sur votre sentiment de compétence, d'auto-efficacité ou de confiance à intervenir auprès de parents qui font face à des difficultés avec leur enfant.

SÉMINAIRES (TRIPLE P - NIVEAU 2)

1. Avez-vous été formé-e à Triple P de Niveau 2 (Séminaires)?

- ❑ Oui
- ❑ Non **(passez à la question 12, page 14)**

2. Avez-vous offert les séminaires Triple P (niveau 2)?

- ❑ Oui
- ❑ Non **(passez à la question 9, page 12)**

3. Combien de séminaires Triple P avez-vous animés au cours de vos 4 dernières semaines de travail?

______ séminaire(s)

4. Combien de séminaires Triple P avez-vous animés depuis votre formation initiale?

______ séminaire(s)

5. En moyenne, combien de personnes assistaient à chaque séminaire ?

- ❑ 10 personnes ou moins
- ❑ 11-20 personnes
- ❑ 21-30 personnes
- ❑ 31-40 personnes
- ❑ Plus de 40 personnes

6. Veuillez indiquer le niveau de difficulté que vous avez éprouvé à animer les séminaires Triple P (encerclez un chiffre entre 1 et 7).

7	6	5	4	3	2	1
Pas difficile du tout		*légèrement difficile*		*Très difficile*		*Impossible*

7. Est-ce que ce niveau de Triple P vous a aidé-e à être plus efficace dans votre travail ? (Encerclez un chiffre entre 1 et 7)

1	2	3	4	5	6	7
Non, définitivement pas		*Non, pas vraiment*		*Oui, relativement*		*Oui, définitivement*

8. Jusqu'à quel point croyez-vous que ce niveau de Triple P a été efficace pour les familles? (Encerclez un chiffre entre 1 et 7)

7	6	5	4	3	2	1
Extrêmement		*Modérément*		*Légèrement*		*Pas du tout*

9. Estimez-vous avoir suivi une formation suffisante pour vous permettre de procéder à des consultations auprès de parents concernant le comportement des enfants? (Encerclez un chiffre entre 1 et 7)

7	6	5	4	3	2	1
Oui, certainement		*Oui, en général*		*Non, pas vraiment*		*Non, pas du tout*

10. Dans quelle mesure vous sentez-vous confiant-e de procéder à des consultations auprès de parents concernant le comportement des enfants? (Encerclez un chiffre entre 1 et 7)

1	2	3	4	5	6	7
Pas confiant du tout		*Pas très confiant*		*Confiant*		*Très confiant*

11. Veuillez évaluer dans quelle mesure vous estimez que vous maîtrisez les compétences suivantes de consultation auprès de parents, sur une échelle de 1 (ne maîtrise pas du tout et a besoin d'aide) à 7 (maîtrise complètement, aucune aide requise).

		1	2	3	4	5	6	7
A	Organiser un environnement propice pour un séminaire sur les pratiques parentales							
B	Installer et utiliser l'équipement audiovisuel requis pour le séminaire							
C	Présenter aux parents un ordre du jour clair du séminaire et le confirmer avec eux							
D	Fournir une justification et une description claire de chaque principe et stratégie utilisés dans le cadre de la série des séminaires							
E	Discuter des causes des problèmes de comportement des enfants							
F	Discuter de stratégies relatives aux pratiques parentales particulières présentées pendant le séminaire							
G	Utiliser des exemples clairs et pertinents afin d'illustrer des principes ou des stratégies clés							
H	Faire des démonstrations des compétences parentales positives particulières présentées pendant le séminaire							
I	Utiliser la répétition du comportement avec un parent volontaire afin de faire la démonstration d'une compétence							
J	Aider les parents à fixer pour leurs enfants des objectifs de changement du comportement particuliers, pouvant être mis en pratique et convenant à l'âge de l'enfant							
K	Gérer la résistance des parents qui assistent au séminaire							
L	Répondre aux questions et aux préoccupations des parents relativement aux informations présentées dans le cadre du séminaire							
M	Avoir recours à la théorie et aux preuves empiriques pour fournir des justifications							

		1	2	3	4	5	6	7
N	Utiliser des techniques de questionnement pour voir si les participants ont compris les points d'apprentissage clés							
O	Déterminer le type et la puissance de l'intervention requise, à partir des commentaires et des questions des parents avant et après le séminaire							
P	Répondre aux questions des parents concernant des problèmes comportementaux ou émotionnels particuliers pendant la période de questions et de réponses ou immédiatement après le séminaire							
Q	Aiguiller les familles vers les organismes appropriés afin qu'elles reçoivent une aide supplémentaire, le cas échéant							

TRIPLE P « CONSULTATIONS PONCTUELLES » (NIVEAU 2) ET « SOINS PRIMAIRES » (NIVEAU 3)

12. Avez-vous été formé-e à Triple P de Niveau 3 (Soins primaires)?

- ❑ Oui
- ❑ Non **(passez à la question 23, page 18)**

13. Avez-vous utilisé les consultations ponctuelles (niveau 2) ou l'intervention individuelle « soins primaires » (niveau 3) de Triple P?

- ❑ Oui
- ❑ Non **(passez à la question 20, page 16)**

14. Avec combien de familles avez-vous utilisé les consultations ponctuelles ou l'intervention individuelle « soins primaires » de Triple P au cours de vos 4 dernières semaines de travail?

______ famille(s)

15. Avec combien de familles estimez-vous avoir utilisé les consultations ponctuelles ou l'intervention individuelle « soins primaires » de Triple P depuis votre formation initiale?

______ famille(s)

16. Au cours des 6 derniers mois, combien de rencontres de niveau 2 (consultation ponctuelle) ou de niveau 3 (soins primaires) avez-vous réalisées chaque semaine, en moyenne?

- ❑ Moins de 1 par semaine
- ❑ 1-2 par semaine
- ❑ 3-4 par semaine
- ❑ 5-6 par semaine
- ❑ 7-8 par semaine
- ❑ 9-10 par semaine
- ❑ Plus de 10 par semaine

17. Veuillez indiquer le niveau de difficulté que vous avez éprouvé à offrir les consultations ponctuelles ou l'intervention individuelle « soins primaires » de Triple P (encerclez un chiffre entre 1 et 7).

7	6	5	4	3	2	1
Pas difficile du tout		*légèrement difficile*		*Très difficile*		*Impossible*

18. Est-ce que ces interventions Triple P vous ont aidé-e à être plus efficace dans votre travail ? (Encerclez un chiffre entre 1 et 7)

1	2	3	4	5	6	7
Non, définitivement pas		*Non, pas vraiment*		*Oui, relativement*		*Oui, définitivement*

19. Jusqu'à quel point croyez-vous que ces interventions Triple P ont été efficaces pour les familles? (Encerclez un chiffre entre 1 et 7)

7	6	5	4	3	2	1
Extrêmement		*Modérément*		*Légèrement*		*Pas du tout*

20. Estimez-vous avoir suivi une formation suffisante pour vous permettre de procéder à des consultations avec les parents concernant le comportement des enfants? (Encerclez un chiffre entre 1 et 7)

7	6	5	4	3	2	1
Oui, certainement		*Oui, en général*		*Non, pas vraiment*		*Non, pas du tout*

21. Dans quelle mesure vous sentez-vous confiant-e de procéder à des consultations avec les parents concernant le comportement des enfants? (Encerclez un chiffre entre 1 et 7)

1	2	3	4	5	6	7
Pas confiant du tout		*Pas très confiant*		*Confiant*		*Très confiant*

22. Veuillez évaluer dans quelle mesure vous estimez que vous maîtrisez les compétences suivantes en matière de consultation avec les parents, sur une échelle de 1 (ne maîtrise pas du tout et a besoin d'aide) à 7 (maîtrise complètement, aucune aide requise).

		1	2	3	4	5	6	7
A	Organiser un environnement propice aux consultations avec les parents							
B	Négocier avec les parents un ordre du jour clair pour chaque consultation							
C	Poser des questions aux parents (y compris obtenir des éclaircissements relatifs au comportement problématique particulier, sa fréquence, son intensité, ses antécédents et les conséquences qui lui sont liées)							
D	Utiliser l'observation directe pour procéder à une analyse des excès et des déficiences du comportement d'un enfant							
E	Choisir des procédures d'évaluation de base fiables et valables							
F	Déterminer le type et l'intensité de l'intervention requise							
G	Communiquer aux parents les résultats de l'évaluation du comportement de l'enfant							
H	Discuter des causes des problèmes de comportement des enfants							

		1	2	3	4	5	6	7
I	Discuter de stratégies relatives aux pratiques parentales							
J	Montrer comment utiliser des compétences parentales positives particulières							
K	Utiliser la répétition du comportement avec les parents pour l'acquisition de compétences							
L	Façonner l'utilisation des compétences parentales par les parents grâce à une rétroaction constructive							
M	Concevoir des stratégies relatives aux pratiques parentales efficaces pour les situations à haut risque, comme lorsque le parent est occupé (p. ex., au téléphone) ou observé par d'autres (p. ex., au magasin)							
N	Aider les parents à fixer pour leurs enfants des objectifs de changement du comportement précis, pouvant être mis en pratique et convenant à l'âge de l'enfant							
O	Gérer la résistance des parents							
P	Répondre aux questions et aux préoccupations des parents concernant la discipline							
Q	Avoir recours à la théorie et aux preuves empiriques pour fournir des justifications							
R	Utiliser l'équipement audiovisuel pendant les consultations avec les parents							
S	Donner aux parents des tâches à domicile (p. ex., tâches de lecture) afin d'encourager l'apprentissage autodirigé							
T	Aiguiller les familles vers les organismes appropriés afin qu'elles reçoivent une aide supplémentaire, le cas échéant							

TRIPLE P DE GROUPE (NIVEAU 4)

23. Avez-vous été formé-e à Triple P de Niveau 4 (Groupe)?

- ❑ Oui
- ❑ Non **(passez à la question 34, page 21)**

24. Avez-vous utilisé le Triple P de groupe (niveau 4) ?

- ❑ Oui
- ❑ Non **(passez à la question 31, page 19)**

25. Avec combien de familles avez-vous utilisé le Triple P de groupe au cours de vos 4 dernières semaines de travail?

______ famille(s)

26. Avec combien de familles estimez-vous avoir utilisé le Triple P de groupe depuis votre formation initiale?

______ famille(s)

27. Au cours des 6 derniers mois, combien de rencontres de groupe avez-vous réalisées chaque semaine, en moyenne?

- ❑ Moins de 1 par semaine
- ❑ 1-2 par semaine
- ❑ 3-4 par semaine
- ❑ 5-6 par semaine
- ❑ 7-8 par semaine
- ❑ 9-10 par semaine
- ❑ Plus de 10 par semaine

28. Veuillez indiquer le niveau de difficulté que vous avez éprouvé à animer les groupes Triple P (encerclez un chiffre entre 1 et 7).

7	6	5	4	3	2	1
Pas difficile du tout		*légèrement difficile*		*Très difficile*		*Impossible*

29. Est-ce que les groupes Triple P vous ont aidé-e à être plus efficace dans votre travail ? (Encerclez un chiffre entre 1 et 7)

1	2	3	4	5	6	7
Non, définitivement pas		*Non, pas vraiment*		*Oui, relativement*		*Oui, définitivement*

30. Jusqu'à quel point croyez-vous que les groupes Triple P ont été efficaces pour les familles? (Encerclez un chiffre entre 1 et 7)

7	6	5	4	3	2	1
Extrêmement		*Modérément*		*Légèrement*		*Pas du tout*

31. Estimez-vous avoir suivi une formation suffisante pour vous permettre d'animer des groupes traitant du comportement des enfants? (Encerclez un chiffre entre 1 et 7)

7	6	5	4	3	2	1
Oui, certainement		*Oui, en général*		*Non, pas vraiment*		*Non, pas du tout*

32. Dans quelle mesure vous sentez-vous confiant-e d'animer des groupes de parents traitant du comportement des enfants? (Encerclez un chiffre entre 1 et 7)

1	2	3	4	5	6	7
Pas confiant du tout		*Pas très confiant*		*Confiant*		*Très confiant*

33. Veuillez évaluer dans quelle mesure vous estimez que vous maîtrisez les aptitudes suivantes de consultation auprès de groupes de parents, sur une échelle de 1 (ne maîtrise pas du tout et a besoin d'aide) à 7 (maîtrise complètement, aucune aide requise).

		1	2	3	4	5	6	7
A	Organiser un environnement propice pour les groupes de parents							
B	Négocier avec les parents un ordre du jour clair pour chaque session en groupe							
C	Utiliser l'observation directe pour procéder à une analyse des excès et des déficiences du comportement d'un enfant							
D	Choisir des procédures d'évaluation de base fiables et valables							
E	Discuter avec un groupe des causes des problèmes de comportement des enfants							
F	Discuter avec un groupe des stratégies relatives aux pratiques parentales							
G	Démontrer le recours à des compétences parentales positives spécifiques							
H	Utiliser la répétition du comportement avec les groupes de parents lors de l'acquisition des compétences							
I	Façonner l'utilisation des compétences parentales par les parents grâce à une rétroaction constructive							
J	Concevoir des stratégies relatives aux pratiques parentales efficaces pour les situations à risque élevé, par exemple lorsque le parent est occupé (p. ex., au téléphone) ou observé par d'autres (p. ex., au magasin).							
K	Aider les parents à fixer pour leurs enfants des objectifs de changement du comportement spécifiques, pouvant être mis en pratique et convenant à l'âge de l'enfant.							
L	Faire face à la résistance des parents au sein d'un groupe.							
M	Répondre aux questions et aux préoccupations des parents concernant la discipline							
N	Avoir recours à la théorie et aux preuves empiriques pour fournir des justifications							

		1	2	3	4	5	6	7
O	Utiliser l'équipement audiovisuel avec les groupes de parents							
P	Donner aux parents des travaux à domicile (p. ex., tâches de lecture) afin d'encourager l'apprentissage autodidacte.							
Q	Gérer les problèmes relatifs au processus au sein du groupe (p. ex., absence de participation, parents qui parlent trop).							
R	Aider les parents à se fixer des objectifs de changement des pratiques ou des comportements qui sont réalistes et spécifiques							
S	Faciliter l'autosuffisance en encourageant les parents à s'auto-surveiller et à auto-évaluer leur performance, par l'entremise de consultations téléphoniques							
T	Recommander les familles à des agences appropriées afin qu'elles reçoivent une aide supplémentaire, le cas échéant.							

TRIPLE P DE CHEMINEMENT (NIVEAU 5)

34. Avez-vous été formé-e à Triple P de Niveau 5 (Cheminement)?

- ❑ Oui
- ❑ Non **(passez à la question 46, page 25)**

35. Avez-vous utilisé le Triple P de cheminement (niveau 5)?

- ❑ Oui
- ❑ Non **(passez à la question 43, page 23)**

36. Avec combien de familles avez-vous utilisé le Triple P de cheminement, en individuel ou en groupe, au cours de vos 4 dernières semaines de travail?

______ famille(s)

37. Avec combien de familles estimez-vous avoir utilisé le Triple P de cheminement, en individuel ou en groupe, depuis votre formation initiale?

______ famille(s)

38. Au cours des 6 derniers mois, combien de rencontres <u>individuelles</u> de cheminement avez-vous réalisées chaque semaine, en moyenne?

- ❑ Moins de 1 par semaine
- ❑ 1-2 par semaine
- ❑ 3-4 par semaine
- ❑ 5-6 par semaine
- ❑ 7-8 par semaine
- ❑ 9-10 par semaine
- ❑ Plus de 10 par semaine

39. Au cours des 6 derniers mois, combien de rencontres <u>de groupe</u> de cheminement avez-vous réalisées chaque semaine, en moyenne?

- ❑ Moins de 1 par semaine
- ❑ 1-2 par semaine
- ❑ 3-4 par semaine
- ❑ 5-6 par semaine
- ❑ 7-8 par semaine
- ❑ 9-10 par semaine
- ❑ Plus de 10 par semaine

40. Veuillez indiquer le niveau de difficulté que vous avez éprouvé à offrir le Triple P de cheminement (encerclez un chiffre entre 1 et 7).

7	6	5	4	3	2	1
Pas difficile du tout		*légèrement difficile*		*Très difficile*		*Impossible*

41. Est-ce que le Triple P de cheminement vous a aidé-e à être plus efficace dans votre travail ? (Encerclez un chiffre entre 1 et 7)

1	2	3	4	5	6	7
Non, définitivement pas		*Non, pas vraiment*		*Oui, relativement*		*Oui, définitivement*

42. Jusqu'à quel point croyez-vous que le Triple P de cheminement a été efficace pour les familles? (Encerclez un chiffre entre 1 et 7)

7	6	5	4	3	2	1
Extrêmement		*Modérément*		*Légèrement*		*Pas du tout*

43. Vous sentez-vous suffisamment formé pour mener des consultations de parents par rapport au comportement d'un enfant? (Encerclez un chiffre entre 1 et 7)

7	6	5	4	3	2	1
Oui, certainement		*Oui, en général*		*Non, pas vraiment*		*Non, pas du tout*

44. Dans quelle mesure vous sentez-vous confiant-e pour mener des consultations avec des parents présentant des cas de maltraitance infantile? (Encerclez un chiffre entre 1 et 7)

1	2	3	4	5	6	7
Pas confiant du tout		*Pas très confiant*		*Confiant*		*Très confiant*

45. Veuillez évaluer votre niveau de compétence par rapport aux aptitudes suivantes nécessaires pour mener des consultations auprès de parents, sur une échelle de 1 (ne maîtrise pas du tout et a besoin d'aide) à 7 (maîtrise complètement, aucune aide requise).

		1	2	3	4	5	6	7
A	Mettre en place un environnement propice à des consultations de parents							
B	Établir avec les parents un ordre du jour clair pour chaque séance							
C	Interroger les parents pour préciser leurs attributions quant au comportement de leur enfant (explications causales)							
D	Interroger les parents pour préciser leurs attributions quant à leur propre comportement (explications causales)							
E	Sélectionner des mesures d'évaluation fiables et valides pour évaluer les effets des interventions de pratiques parentales axées sur la colère parentale							
F	Aider les parents à découvrir le lien entre leurs propres pratiques parentales et la façon dont ils ont été élevés en tant qu'enfant							

		1	2	3	4	5	6	7
G	Aider les parents à identifier leurs attentes à l'égard de leurs enfants							
H	Inciter les parents à fixer des objectifs de changement de comportement spécifiques, réalisables et adaptés à l'âge pour leurs enfants							
I	Traiter les questions et les préoccupations des parents à propos des conseils en matière de pratiques parentales et des méthodes disciplinaires							
J	Aider les parents à fixer des objectifs réalistes et spécifiques de changement de pratiques/comportement							
K	Fournir une conceptualisation cognitive aux parents sur la façon dont leurs émotions fonctionnent							
L	Discuter sur la façon dont les émotions des parents affectent leurs pratiques parentales							
M	Aider les parents à identifier leurs schémas de pensées/mode de pensée dysfonctionnels							
N	Aider les parents à interrompre, commuter, combattre ou contester les énoncés ou pensées attributionnels négatifs							
O	Aider les parents à concevoir des énoncés d'adaptation à la colère							
P	Présenter et entraîner les parents sur l'utilisation des stratégies de relaxation							
Q	Aider les parents à concevoir des plans d'adaptation personnels pour des situations potentiellement stressantes							
R	Donner aux parents des devoirs appropriés à faire à la maison (ex. lectures, activités à faire avec leur enfant) en utilisant une approche d'autorégulation							
S	Orienter les familles à risque de maltraitance vers les organismes adéquats si une aide est nécessaire							

SOUTIEN

46. Au cours des derniers 6 mois, combien de fois avez-vous consulté d'autres praticiens Triple P pour obtenir des conseils, des suggestions ou du soutien dans l'utilisation du programme?

- ❑ Jamais
- ❑ 1-2 fois
- ❑ 3-4 fois
- ❑ 5-6 fois
- ❑ 7-8 fois
- ❑ 9-10 fois
- ❑ Plus de 10 fois

47. Jusqu'à quel point votre milieu de travail soutient-il l'utilisation de Triple P?

- ❑ Très soutenant
- ❑ Plutôt soutenant
- ❑ Pas très soutenant
- ❑ Pas soutenant du tout

48. Quelle est l'attitude générale vis-à-vis de Triple P dans votre milieu de travail?

- ❑ Très positive
- ❑ Plutôt positive
- ❑ Plutôt négative
- ❑ Très négative

49. Jusqu'à quel point cette attitude générale influence votre utilisation de Triple P?

- ❑ Beaucoup
- ❑ Modérément
- ❑ Un peu
- ❑ Pas du tout

50. Au cours des 6 derniers mois, à quelle fréquence vous a-t-on offert de la supervision ou du soutien par les pairs en lien avec Triple P?

- ❑ Une fois par semaine ou plus
- ❑ Plus d'une fois par mois
- ❑ Une fois par mois
- ❑ Chaque 2-3 mois
- ❑ Chaque 4-5 mois
- ❑ Une seule fois en 6 mois
- ❑ Jamais

51. Sur une échelle de 1 à 5, quel est votre niveau de satisfaction vis-à-vis de la supervision / du soutien par les pairs que vous avez reçus ? (jusqu'à quel point cela a été utile pour votre utilisation de Triple P?)

1	2	3	4	5
Très insatisfait-e				*Extrêmement satisfait-e*

52. Sur une échelle de 1 à 5, jusqu'à quel point les discussions entre collègues ont-elles été aidantes?

1	2	3	4	5
Pas du tout aidantes				*Extrêmement aidantes*

❑

Avez-vous des commentaires à nous transmettre en lien avec votre expérience du programme Triple P?

	Jour	Mois	Année
**Date de complétion du questionnaire :**			

Veuillez SVP nous retourner ce questionnaire complété le plus rapidement possible.

Merci de votre collaboration !
